# Dr. Noe’s Case in June Number

**Published:** 1891-09

**Authors:** A. T. Noe

**Affiliations:** Craig, Burt County, Nebraska; formerly; Bethany Heights, Lincoln, Nebraska


### DR. NOE'S CASE IN JUNE NUMBER.

EDITORS OF THE HOMŒOPATHIC PHYSICIAN:

Since writing up that case you reported for me in the June number, on page 254, I have received several replies from all over the United States, and all of them from homœopaths. The remedies suggested are: Hyos., Nux-v., and Lach. I would like to say to my readers that I have used all of the above remedies in all the potencies, and waited, thinking every time I had the remedy. But no improvement. I have given Sepia high and it removed her corns last spring, but it fails this spring. She has not that all-gone feeling in the stomach, however, and is made better by eating.

I will add some more valuable symptoms which may enable the readers to see more clearly the red string of the case:

Every spring and summer her feet trouble her. Her feet become so cramped that she has to pull off her shoes. She is worse sitting down or riding. Corns on toes and balls of feet, hard, horny, and painful. She complains much of the pain they cause her. She says her feet are always damp when she removes her shoes at night, but it is not noticeable during the day. Her feet are warm or hot at night during warm weather, and cold in winter time. She has had Sulphur, but no improvement that would last over two months, and I have used the CM potency. Riding in the cold wind gives her the headache.

When she has some of these headaches, her headache would come on with a numb feeling in her fingers, face, and tongue, and some blindness. After lying down for an hour or so then the numbness would pass off and headache would come on. She would have to go into her bedroom and lie down and cover her head up and go to sleep. If she was enabled to sleep an hour or two she would wake up with her headache much better, but with sense of soreness of head. The head symptoms are made better by warmth, which makes one think of Nux-v. and Sil.

She says she would rather do anything than to try to make a dress for herself. She gets so angry and nervous that she can't do anything, and is very rough at any disturbance the children make.

The palms of her hands perspire when sewing so that she is wiping them on her apron every few minutes. She is very sensitive to the least thing. If she has a splinter in her finger she can hardly bear the thought of picking it out, but she can stand by and assist in performing an operation on any one else without any fear or without getting nervous. She has in-growing toe-nails; has always had them. Had one cut out last year. She has much musty smelling perspiration in armpits. She cannot take a cold bath, it chills her so. Must have warm water. She starts at least unexpected noise or on sudden appearance of any one whom she knows or any member of her family so that she is weak and nervous for an hour.

She is stout enough to do all of her work and feels very well at times. Easily excited from things unexpected, not from things that are expected.

There is no lacerated cervix, but a laceration of the perinæum of one-half inch, which I don't think gives her any trouble.

She menstruates regularly and normally as far as I can learn. Her nose gets sore every time she menstruates. Nose feels as if a boil was coming in alæ nasi, they get red and thicken up. Keeps her all the time picking at nose. When she returns home from visiting, though no one has come to the house in her absence, she will look in every room and in closets and under beds before she is satisfied. Says she fears there must be some one in the house that will hurt her or kill her and children.

She is very irritable when busy at work, and was constipated during winter. Has taken no allopathic treatment for over ten years.

Has had gall-stone colic, and passed a few gall-stones four and one-half years ago, but none since. Her father used to be troubled with gall-stones. She sleeps well when all are at home, but jumps in sleep when she has been doing something that is straining on nerves day previous. But when alone with her two children can't sleep because she fears there is a man about the house. She is scared from the least noise she may hear.

I believe I have given the case in full, and would be pleased to have any further suggestion regarding this case, for I have been trying two years to cure her, but without success.

A. T. Noe, M. D.,

Craig, Burt County, Nebraska; formerly
Bethany Heights, Lincoln, Nebraska.

